# *Nrf2* and Cardiovascular Defense

**DOI:** 10.1155/2013/104308

**Published:** 2013-04-04

**Authors:** Reuben Howden

**Affiliations:** Laboratory of Systems Physiology, Department of Kinesiology, University of North Carolina at Charlotte, Charlotte, NC 28223, USA

## Abstract

The cardiovascular system is susceptible to a group of diseases that are responsible for a larger proportion of morbidity and mortality than any other disease. Many cardiovascular diseases are associated with a failure of defenses against oxidative stress-induced cellular damage and/or death, leading to organ dysfunction. The pleiotropic transcription factor, nuclear factor-erythroid (NF-E) 2-related factor 2 (*Nrf2*), regulates the expression of antioxidant enzymes and proteins through the antioxidant response element. *Nrf2* is an important component in antioxidant defenses in cardiovascular diseases such as atherosclerosis, hypertension, and heart failure. *Nrf2* is also involved in protection against oxidant stress during the processes of ischemia-reperfusion injury and aging. However, evidence suggests that *Nrf2* activity does not always lead to a positive outcome and may accelerate the pathogenesis of some cardiovascular diseases (e.g., atherosclerosis). The precise conditions under which *Nrf2* acts to attenuate or stimulate cardiovascular disease processes are unclear. Further studies on the cellular environments related to cardiovascular diseases that influence *Nrf2* pathways are required before *Nrf2* can be considered a therapeutic target for the treatment of cardiovascular diseases.

## 1. Introduction

Cardiovascular diseases contribute more to morbidity and mortality than any other group of diseases in the developed world [1]. Oxidative stress is an important component in the pathogenesis of many cardiovascular disorders, including atherosclerosis [2, 3], hypertension [4], heart failure [5], and ischemia/reperfusion injury [6–8]. Sources of potentially damaging reactive oxygen species (ROS) leading to oxidative stress have been extensively reviewed (e.g. [9–11]) and include, but are not limited to, mitochondrial electron transport chain inefficiencies, NADPH oxidase and ubiquitous xanthine oxidase activity, and metallic ions released during cell lysis. This suggests that the activation of antioxidant defenses has an important role in reducing oxidant-induced cellular damage. However, cellular damage or death can still result, leading to organ dysfunction, when cellular antioxidant defenses are overwhelmed by excess ROS production [12].

A well-established and critical component to cellular antioxidant defense mechanisms is expression of direct ROS scavenging enzymes, phase II detoxification enzymes, and other detoxification proteins bearing antioxidant response elements (AREs) in their promoter regions. A principal regulator of the ARE is the highly conserved transcription factor nuclear factor-erythroid (NF-E) 2-related factor 2 (NRF2 for human, *Nrf2* for mouse and rat), which is a member of the Cap “n” collar family of transcription factors (more details on *Nrf2* are provided elsewhere in this special issue). *Nrf2* induces transcriptional activation of a number of ARE-bearing antioxidants, including NAD(P)H dehydrogenase (quinone 1) (NQO1), superoxide dismutases (SODs), and glutathione peroxidases (GPx). Many of the *Nrf2* regulated enzymes are essential in the pathogenesis of cardiovascular diseases [13]. However, there exists evidence for both beneficial and detrimental effects of *Nrf2* activation in the cardiovascular system. Further investigation is required to better understand the range of interactions between *Nrf2* and the cardiovascular system, which could have profound effects on the pathogenesis of cardiovascular diseases. Therefore, the purpose of this review is to discuss the current evidence for a role of *Nrf2* in a selection of prominent cardiovascular pathologies.

## 2. *Nrf2* and Atherosclerosis

Several vascular disease processes are associated with oxidative stress, and therefore suboptimal antioxidant defenses may increase patient risk and accelerate disease progression [14]. Atherosclerosis is an inflammatory disease [15] characterized by endothelial infiltration and accumulation of oxidized low-density lipoproteins (LDLs), physical damage to the endothelium (e.g. turbulent blood flow, hypertension, and/or toxins from cigarette smoking), and/or infection (e.g. HIV). This process leads to atherosclerotic lesions, compromised blood vessel diameter, and increased risk of ischemia, which is a major concern especially in the myocardium.

Interestingly, evidence suggests that susceptibility to atheroma formation is not uniform throughout the vascular system. Several studies suggest that shear stress generated by oscillatory, nonunidirectional, and turbulent blood flow, for example at bifurcations or points of vessel branching, results in atheroma-prone regions [16, 17], increasing the risk of atherosclerosis development. Conversely, atheromas are less likely to form in vascular regions only exposed to unidirectional laminar blood flow [16, 18]. It is well established that laminar vascular wall shear stress stimulates the release of nitric oxide (NO), known for its protective role against atherosclerosis (see [19] for review). However, when blood flow becomes oscillatory (e.g. high flow rates, stenosis, or vessel branching), shear stress on the vascular wall is inconsistent. This reduces NO production and increases superoxide release, which leads to enhanced oxidative stress and atherosclerosis progression [20]. Therefore, laminar versus oscillatory blood flow may be responsible for the apparent confusion regarding the role of *Nrf2* in the pathogenesis of atherosclerosis. It is becoming clear that laminar blood flow promotes antiatherogenic activation of *Nrf2,* and oscillatory blood flow suppresses *Nrf2* activation, creating a proatherogenic environment [21, 22]. However, specific blood flow characteristics, in relation to atherosclerosis progression, should be investigated in greater detail to improve the understanding of the interaction between blood flow, *Nrf2,* and atherosclerosis susceptibility.

## 3. *Nrf2* as an Antiatherogenic Factor

It is becoming increasingly apparent that *Nrf2* is important to vascular integrity and long-term endothelial function, for example, sustained release of NO and protection from apoptosis [23–29]. Conversely, specific changes in vascular physiology that are related to *Nrf2* can lead to increased susceptibility to atheroma development, such as increases in oxidative stress leading to oxidation of LDLs, reduced NO production, and increased levels of superoxide [20].

One important stage of atherosclerotic plaque formation is a well-established endothelial infiltration by macrophages and foam cell formation following macrophage absorption of accumulated LDLs. In mice, *Nrf2* is an important component in this process, since macrophages exposed to oxidized LDLs (oxLDL) increased *Nrf2* expression in response, which indirectly protected macrophages from oxLDL-mediated injury via phase II antioxidant enzyme activity [30]. Moreover, absence of *Nrf2* in high fat diet myeloid-derived macrophages [31] or LDL receptor deficient myeloid-derived macrophages [32] increased foam cell formation and atherosclerosis progression, further suggesting that *Nrf2* is important in resistance to atherosclerosis.

Increases in *Nrf2* expression at this stage of atherosclerosis development is significant because of downstream effects on heme oxygenase-1 (HO-1), which produces antiatherogenic reductions foam cell formation [33–36]. Moreover, atherosclerosis was accelerated in HO-1 absent/apolipoprotein E-deficient (*ApoE* ^−/−^) mice [37]. The *ApoE* ^−/−^ mouse strain is a well-established model for atherosclerosis [38, 39]. In addition to its antioxidant properties, HO-1 protects against inflammation in vascular tissue [33, 40, 41] and has been reported to act in an atheroprotective manner through this mechanism [42, 43]. Since oxidative stress and inflammation are known to be important at all stages of atherosclerosis development [2, 44], these data suggest a central role for HO-1 in atherosclerosis pathophysiology. 

In mice, HO-1 has also been reported to suppress atherosclerotic lesion formation by reducing oxLDL-induced transmigration of monocytes, and the reverse was found when HO-1 was inhibited [34]. In addition to oxLDL activated increases in HO-1 expression through *Nrf2*, other *Nrf2* downstream targets appear to play a role in this process, like glutathione-cysteine ligase modifying subunit and NQO1, both of which have been associated with protection against atherosclerosis [26]. In adolescents, low serum glutathione was an independent risk factor for parental coronary heart disease risk [45]. Moreover, low GPx levels, for which glutathione is a cofactor, combined with low high-density lipoprotein levels may be partly responsible for increased atherosclerosis-related mortality rates in humans [46]. *Nrf2* was identified as an important regulator of GPx in mice [47], and therefore taken together, these data demonstrate that *Nrf2* is an important component in protection against the pathogenesis of atherosclerosis.

HO-1 may also offer protection from atherosclerosis-related morbidity and/or mortality at more advanced stages of the disease by promoting atherosclerotic plaque stability. It has been suggested that matrix metalloproteinase 9 (MMP9) levels are linked to plaque destabilization [48, 49], which are important events in acute constriction of vessel blood flow and sudden cardiac or cerebral events. Interestingly, an atheroprotective role for HO-1 may be partially associated with MMP9 suppression to maintain or improve plaque stability [50], potentially avoiding an acute, life-threatening coronary or cerebral event. These data present convincing evidence for the importance of *Nrf2* and its downstream targets in protection against atherosclerotic plaque formation or stability.

In addition to atherosclerotic processes, *Nrf2* expression may also be induced by extrinsic factors, leading to protection against the disease. For example, activation of *Nrf2* by dosing mice with the cruciferous vegetable extract sulforaphane had an anti-inflammatory effect on atherosusceptible endothelial cells [51], although the effect of the sulforaphane dose used in their study on *Nrf2* expression was not assessed. Therefore, it is possible that in this case, *Nrf2* was not activated and sulforaphane induced other anti-inflammatory factors. However, it should be noted that sulforaphane has been reported to induce expression of antioxidant enzymes regulated by *Nrf2 *[52–54]. Furthermore, increased *Nrf2* message and nuclear NRF2 were found with sulforaphane-treated mice in a model of respiratory syncytial virus disease [55].

## 4. *Nrf2* as a Proatherogenic Factor

Interestingly, *Nrf2* has been reported to be proatherogenic in an elegant study comparing atherosclerotic plaque formation in *ApoE* ^−/−^ mice that were either *Nrf2* sufficient or deficient, combined with either a 10- or 20-week high fat diet [56]. *ApoE* ^−/−^ *Nrf*2^−/−^ mice developed significantly less aortic plaque area and loss of vessel wall elasticity compared to *ApoE* ^−/−^ *Nrf*2^+/+^ mice, which was reported to occur in a sex-dependent manner [57]. Moreover, this effect appeared to be partly dependent on diet, which increases the urgency for diet modification in the general population, especially in low socioeconomic regions where diet-related susceptibility to atherosclerosis, among other cardiovascular diseases, is known to be higher [58]. However, the study of Barajas et al. suggests independent actions of *Nrf2* and HO-1 in atherosclerosis. In *ApoE* ^−/−^mice, *Nrf2* deletion resulted in atherosclerosis suppression [57], but with HO-1 deletion the atherosclerosis was accelerated [37]. Considering the regulation of HO-1 by *Nrf2*, this illustrates the current confusion regarding the role of *Nrf2* in atherosclerosis.

The paradox between compromised antioxidant defenses in *Nrf*2^−/−^ mice and lower aortic atheroma area could be associated with a reduction in macrophage uptake of oxLDL and foam cell formation, as previously discussed. The scavenger protein CD36 regulates macrophage uptake of oxLDL, and normal oxLDL-induced increases in CD36 expression were not found in *Nrf*2^−/−^ macrophages [59]. This suggests that in atherosclerosis development, inhibition of macrophage uptake of oxLDL is more important than antioxidant capacity, both of which are regulated by *Nrf2*. This highlights the current confusion regarding the role of *Nrf2* in atherosclerosis development. In the previous section, discussion of *Nrf2* as an antiatherogenic factor involved increases in *Nrf2* expression leading to lower foam cell formation in the presence of HO-1 [33, 35, 36, 60]. However, these opposing influences of *Nrf2 *on atherosclerosis development operated via different mechanisms and the degree of interindividual atherosclerosis patient difference in the prominence of one mechanism or the other are not known but may be critical in understanding the progression of this disease.

In addition to *Nrf2*-mediated upregulation of scavenger proteins promoting atherosclerosis progression, other factors regulated by *Nrf2* may add to a proatherosclerotic effect. Activating transcription factor 4 (ATF4), known to control vascular endothelial growth factor, stimulates plaque formation by recruiting monocytes to the atherogenic region [61, 62]. Recently, crosstalk between *Nrf2* and ATF4 was demonstrated in endothelial cells [63], further suggesting a proatherogenic effect of *Nrf2*. However, it is beyond the scope of this review to discuss all factors that interact with *Nrf2* in the pathogenesis of atherosclerosis, principally because *Nrf2* is a highly influential gene, especially when enhanced oxidative stress is present. For example, *Nrf2* is known to interact with other well-established pro-atherogenic factors, including vascular cell adhesion molecule 1 [64], NQO1 [65], and interleukin-1 [66]. However, it should be noted that NQO1 has also been reported as both an anti- and proatherogenic factor and therefore improved the understanding of the role of NQO1 in atherosclerosis susceptibility that may lead to clarification of *Nrf2* influences on this process. Nonetheless, it is possible through multiple mechanisms that *Nrf2* produces competing effects on the pathophysiology of atherosclerosis, which highlights the complexity of this disease.

## 5. *Nrf2* and Ischemia-Reperfusion Injury

It has long been recognized that compromised blood flow and cellular perfusion leading to ischemia has major injurious effects on the organ in question. Prominent examples include stroke, myocardial infarction, and organ transplantation. Therefore, reestablishment of blood flow as quickly as possible is a primary clinical goal in ischemia. However, an acute restoration of blood flow to an ischemic region can lead to an enhanced degree of injury as a consequence of oxidative stress compared to the initial period of ischemia.

The myocardium is particularly vulnerable to ischemic injury, because oxygen uptake at any given time during cellular perfusion is around 80%, and therefore cardiac myocytes are unable to significantly increase percent oxygen uptake from arterial blood when blood flow is severely compromised by vascular constriction (e.g. atherosclerotic plaque thrombosis or vasospasm, leading to ischemia). When blood flow is restored however, a substantial inflammatory response is induced [67], significantly increasing oxidative stress, which can overwhelm antioxidant defenses resulting in cardiac dysfunction from cell damage or death. This process makes *Nrf2* an important candidate for resistance to ischemia-reperfusion injury, but there is little information about its role in this situation.

Nonetheless, in rat cardiac H9c2 cells, simulated ischemia reperfusion (10 hrs hypoxia, followed by 16 hrs normoxia) resulted in a significant increase in intracellular ROS levels. Under the same conditions, H9c2 cells were treated with the phase II antioxidant enzyme inducer D3T, which was accompanied by a significant reduction in intracellular ROS levels. In these cells, increases in *Nrf2* mRNA and protein were found, suggesting that *Nrf2* may be important in controlling intracellular ROS levels following ischemia reperfusion [68]. Conversely, in rat hearts, 30 minutes of left anterior descending coronary artery occlusion resulted in a reduction in *Nrf2* nuclear protein, which was prevented by ischemic preconditioning of the myocardium [69]. This finding is very important as it suggests that in order for *Nrf2* to initiate antioxidant defenses against reperfusion-induced oxidative stress, the length of the prior ischemic phase may be a critical factor. Early rescue from ischemia may attenuate *Nrf2* responses to oxidative stress upon reperfusion, reducing protection from reperfusion-induced oxidative stress. Alternatively, ischemic preconditioning may act as an “early warning” signal and activate *Nrf2* prior to a prolonged ischemic event. Acute activation of *Nrf2* has been shown as cardioprotective following ischemia reperfusion. When mice were treated with hydrogen sulfate [70] or 4-hydroxy-2-nonenal [71] to activate *Nrf2* prior to cardiac ischemia reperfusion, reduced infarct size *in vivo* or improved recovery time in Langendorff-perfused mouse hearts were observed respectively.

## 6. *Nrf2* and Hypertension

While oxidative stress and hypertension appear to be related, a “chicken or the egg” scenario means that it is not clear if oxidative stress is a contributing factor to hypertension or if hypertension induces oxidative stress, even though both are likely the case. There are some convincing arguments for the latter [72], although it is possible that oxidative stress caused by preexisting disease (e.g. diabetes) could be a catalyst for hypertension [73]. Certainly, increased levels of ROS in renin-angiotensin-induced hypertension have been established [74, 75]. 

NADPH oxidases (NOX for human, Nox for mouse) are a significant source of ROS in cardiovascular diseases, including angiotensin II-dependent hypertension [74, 76, 77]. A number of Nox isoforms are emerging as important components in the pathophysiology of hypertension in their interaction with *Nrf2*. Nox1, expressed by vascular smooth muscle cells, has been reported to stimulate an increase in ROS levels during an angiotensin II-mediated pressor response [78]. Moreover, activation of *Nrf2* by Nox1 has been found in response to intermittent hypoxia [79], suggesting a mechanism to attenuate oxidative stress through increases in *Nrf2* expression. Vascular endothelial cells express Nox2, and increases in NOX2 levels have recently been associated with angiotensin II-mediated hypertension, endothelial dysfunction, and vascular remodeling [80]. These data suggest that increases in Nox are an important mechanism for resistance to oxidative stress in hypertension mediated by angiotensin II dysfunction.

*Nrf2* may also be important in blood pressure regulation through an alternative and interesting mechanism. *Nrf2* induces expression of HO-1, which has hypotensive effects when upregulated in spontaneously hypertensive rats [81–83]. HO-1 is also implicit in the production of carbon monoxide (CO), in the breakdown of heme into CO, iron, and bilirubin. CO has direct vasodilatory effects [84], which appear to be independent of NO [85]. CO also inhibits the production of endothelin [86], a powerful vasoconstrictor, which is likely an important component of CO effects on vascular tone, regulated by HO-1, the expression of which is induced by *Nrf2*. Moreover, a number of studies have shown reduced blood pressure in response to increases in HO/CO pathway activity in spontaneously hypertensive rats [87–89]. While speculative, these data suggest that *Nrf2* may be important in blood pressure regulation in a capacity other than its part in antioxidant defenses.

However, the potential role for *Nrf2* regulation of HO-1 in blood pressure control is not well defined and may only become important under specific conditions of oxidative stress, like exposure to lipopolysaccharide [90]. Moreover, there were no differences in basal blood pressure between *Nrf*2^−/−^ and wild-type (WT) mice [91]. Li et al. also reported no significant differences between *Nrf*2^−/−^ and WT mice in angiotensin II induced blood pressure elevation. However, the 16 mmHg greater response in systolic blood pressure in WT mice should not go unnoticed from a clinical perspective, suggesting a potential inhibition of hypertensive responses to angiotensin II in *Nrf*2^−/−^ mice.

*Nrf2* expression was upregulated in deoxycorticosterone acetate (DOCA)-salt-induced hypertension in rats. This response, which was enhanced by concomitant epicatechin treatment (*Nrf2* inducer), attenuated the hypertensive response [92]. However, it seems likely that this was due to increases in oxidative stress in association with hypertension, rather than a direct effect of *Nrf2* on blood pressure regulation.

It is clear that *Nrf2* is important, either directly or indirectly, in blood pressure regulation under specific biological environments (e.g. hypertension). However, the circumstances in which *Nrf2* influences blood pressure must first be described in detail before *Nrf2* can be considered as a target for blood pressure therapy in the clinical setting.

## 7. *Nrf2* and Heart Failure

Increased oxidative stress in the diseased myocardium is a well-established phenomenon. Therefore, the potential for *Nrf2* being an important factor in either prevention or slowing of pathophysiologic processes in the myocardium is high. In relation to heart failure, ROS impair cardiac function [93] and increase susceptibility to arrhythmia [94] by a direct toxic effect of increased necrosis and apoptosis [95].

Several *Nrf2* downstream target genes have been associated with protection against abnormal myocardial remodeling in response to hypertension, including HO-1 [96, 97], SOD [98], and GPx [99]. Unfortunately, the role of *Nrf2* in heart failure, while likely on the evidence, has not received significant attention. However, some evidence suggests that *Nrf2* is protective against pathological myocardial hypertrophy and heart failure. In a mouse model of pressure overload by transverse aortic constriction, *Nrf2* overexpression attenuated ROS production and hypertrophic growth in cardiomyocytes, and cardiac fibroblasts [100]. This protective effect of *Nrf2* in myocardial remodeling and heart failure may be mediated through Nox4 [101], which is known to be an important regulator of reduction-oxidation (redox) signaling in many cell types including cardiomyocytes and is a major source of mitochondrial oxidative stress during pressure overload [102]. Furthermore, recent studies have demonstrated cardioprotective activation of *Nrf2* by the Krebs cycle intermediate fumarate [103] and triterpenoids [104–106], suggesting potentially useful treatments with fumarate derivatives or triterpenoids in patients suffering from pathological levels of oxidative stress.

However, while acute activation of *Nrf2* is cardioprotective [70, 71, 107], there is accumulating evidence that chronic activation of *Nrf2* may be harmful to cardiac function [108, 109] leading to pathophysiological processes and heart failure. Chronic activation of *Nrf2* has been reported in association with the concept of “reductive stress” in the murine cardiac hypertrophy and heart failure model of human *α*B-crystallin overexpression [108]. In this model, constitutive activation of *Nrf2* has been reported due to an excess of the reducing equivalents, reduced GSH and NADPH. Therefore, more information about the dynamics of acute versus chronic *Nrf2* activation is required before useful treatment strategies taking advantage of this mechanism can be developed.

Moreover, there are potentially important comorbidity effects that are associated with increases in oxidative stress in the myocardium that point to *Nrf2* playing a protective role. For example, cardiac myocyte insulin resistance is a key component to diabetes-induced cardiac dysfunction, and oxidative stress can exacerbate this scenario. *Nrf2* expression was suppressed in diabetic mice with cardiomyopathy in late stage disease [110]. In the same study, oxidative stress in cardiomyocytes (HL-1 cells) led to depressed *Nrf2* expression, extracellular signal-related kinase (ERK) activation, and a lower glucose metabolism.

This novel interaction between diabetes-related cardiomyopathy and *Nrf2* could provide insight into individual susceptibility to diabetic complications in the cardiovascular system and therefore should be investigated carefully. The principle reason for this need is the current confusion regarding the influence of ERK during cellular stress. ERK signaling is important in the activation of *Nrf2* in response to oxidative stress [111]. Several studies reported a protective effect of oxidative stress-induced ERK activation [112–117], which is not surprising considering its importance in *Nrf2* signaling. Conversely, other studies found that oxidative stress-induced upregulation of ERK was a contributing factor to apoptosis [115, 118, 119]. The precise mechanism by which ERK stimulates or prevents apoptosis is not clear. However, considering the important role of ERK in normal cell division [120], ERK-induced apoptosis has been suggested to be a mechanism to prevent uncontrolled cell proliferation under certain conditions of oxidative stress (e.g. cancer) [121], which could have negative implications when oxidative stress (e.g. hyperoxia) is not accompanied by aberrant cell proliferation. 

## 8. *Nrf2*, Age, and Cardiovascular Disease

The Nrf2-Keap1 pathway is a critical element to redox homeostasis in the myocardium [109, 122]. With age, expression of several *Nrf2 *downstream targets declined in rats [123], and age-related arterial *Nrf2* dysfunction in *Macaca mulatta *[124] and rats [125] has been reported. Since about 75% of cardiovascular disease associated deaths occur in people over the age of 65 years [126] and the mean global population age is increasing, this presents a significant public health concern. Especially important to consider is the large number of diseases associated with oxidative stress, not least cardiovascular diseases.

However, it may be possible to resist the reduction in *Nrf2* activity associated with the aging process. In young mice, expression of myocardial *Nrf2* and several downstream antioxidant target genes have been demonstrated to increase significantly after treadmill exercise comprising 90 minutes per day for 2 days [122]. Moreover, age-related reductions in *Nrf2* transcriptional activity in the myocardium were reversed in mice subjected to the same treadmill exercise or following 6 weeks of moderate treadmill exercise training [127]. Exercise-induced increases in *Nrf2* activity were accompanied by increased levels of antioxidants, like NQO1, HO-1, and GPx1 with corresponding attenuation of ROS status in both young and aged mice [127]. These data highlight the potential importance of habitual exercise to maintain *Nrf2* function in an aging population.

Two important methods by which *Nrf2* activity can be maintained or restored in the myocardium during the aging process are apparent in the literature. First, as is frequently reported, habitual exercise is beneficial for reducing risk of a host of diseases, not least cardiovascular disease and therefore represents a simple, nonpharmacological intervention to protect against age-related *Nrf2* dysfunction. Second, there is a significant amount of evidence suggesting *Nrf2* as a useful therapeutic target to treat oxidative stress related diseases.

## 9. Models for Assessing Cardiopulmonary Responses to Oxidative Stress in Rodents

Exposure of mice to high concentrations of oxygen in air (hyperoxia) for prolonged periods (3–5 days) induces significant oxidative damage to the lung similar to important diseases like acute lung injury (ALI), the more severe acute respiratory distress syndrome (ARDS), and bronchopulmonary dysplasia (BPD). Moreover, significant effects of this model on the cardiovascular system are becoming apparent. In many studies, mice or rats were group housed in exposure chambers (e.g. [128–132]), or standard rodent cages were placed together in large exposure chambers (e.g. [133, 134]). This has been a successful and cost-effective method for exposing multiple animals simultaneously to investigate responses and adaptations in many biological systems, but principally in the lung. Prolonged exposure of mice to hyperoxia leads to acute alveolar inflammation and pulmonary epithelial and endothelial barrier necrosis leading to pulmonary edema and progressively compromised pulmonary gas exchange [135], which has significant effects on cardiac function [136]. 

 Cardiovascular and pulmonary function in conscious, freely moving rodents can be monitored in real time. Electrocardiogram, blood pressure, electroencephalogram, body temperature, and activity level waveforms can be recorded continuously in animals using implantable radio-telemetry transmitters, and pulmonary function can be recorded using whole body plethysmography. Recently, combination of these methods was used to investigate the genetic component to cardiopulmonary function in a wide range of commercially available strains of mice [137]. In order for these systems to work correctly, mice must be singly housed, and therefore previous exposure chamber arrangements with group housed animals would not be appropriate for radio-telemetry or whole body plethysmography. Whole body plethysmographs have been used as hyperoxia exposure chambers (as well as a wide range of inhalants) using mice implanted with ECG telemeters, creating a model for assessing continuous cardiopulmonary responses to hyperoxia (also possible for other species, e.g. rats or rabbits) [136]. This model is a very useful tool for assessing cardiopulmonary responses to hyperoxia-induced oxidative stress and was successfully implemented to investigate the role of *Nrf2* in cardiopulmonary responses to oxidants, included in this issue (refer to Howden et al.).

## 10. Conclusions

*Nrf2* is a key component to cellular redox homeostasis in the attenuation of oxidative stress-associated pathological processes. In the cardiovascular system, patients with insufficient NRF2 levels in multiple tissues are likely susceptible to several adverse components of disease development. If *Nrf2* expression is insufficient to protect against hypertension, then NRF2 is likely insufficient to protect against the resultant oxidative stress, atherosclerosis, and heart failure, highlighting the urgency for investigating *Nrf2* further as a potential therapeutic target. Alternatively, there is evidence for increases in *Nrf2* activity being detrimental to disease resistance and/or accelerating pathogenesis in cardiovascular diseases. 

For example, while activating *Nrf2* shortly before initiating ischemia reperfusion may be beneficial in terms of cardiac function outcome, there is some evidence that chronic activation of *Nrf2* could be detrimental to cardiac function. Therefore, further work is required to understand the role for *Nrf2* in cardiovascular pathogenesis before *Nrf2* can be seriously considered as a therapeutic target for treatment of cardiovascular diseases. This is especially important when considering the increasing prevalence of multiple comorbidities in aging populations [138]. 
